# Serum Zinc Level in Patients with Severe Genital Warts: A Case-Control Study in a Dermatology Hospital

**DOI:** 10.1155/2022/7616453

**Published:** 2022-08-02

**Authors:** Thuy Nguyen Dac Luong, Chuyen Thi Hong Nguyen, Al-Niaimi Firas, Trung The Van

**Affiliations:** ^1^Department of Dermatology, University of Medicine and Pharmacy at Ho Chi Minh City, Vietnam; ^2^Department of Dermatology, Alborg University, Aalborg, Denmark

## Abstract

**Background:**

Genital warts are a common sexually transmitted disease (STD), and there is no method that completely prevents its recurrence. Recently, zinc has been used in the treatment of cutaneous warts. Nondestructive action, ease of use, and promising results with low chances of relapse were reflected in the treatment. These effects may arise from the immunomodulatory activity of zinc in the event of a viral infection.

**Objectives:**

This study was aimed at identifying the relationship between the serum zinc level and the clinical characteristics of patients with genital warts.

**Materials and Methods:**

A case-control study was conducted. Genital warts were diagnosed by clinical examination, and disease severity was demonstrated based on the number of affected sites or the spread of lesions. The serum zinc level was measured using atomic absorption spectrophotometry.

**Results:**

A total of 78 patients with genital warts and 78 healthy volunteers were enrolled in the study. The mean serum zinc level in the genital wart group was lower than that in the control group (81.83 ± 13.99 *μ*g/dL vs. 86.66 ± 17.58 *μ*g/dL); however, this difference was not statistically significant (*P* > 0.05). The mean concentrations of serum zinc in patients having more than one affected site, spread > 2 cm^2^, or ten or more lesions were significantly lower than those of the control group (*P* < 0.05).

**Conclusions:**

The results suggested that severe genital warts may be associated with a low serum zinc level in patients.

## 1. Introduction

Human papillomaviruses (HPV) are a group of deoxyribonucleic acid (DNA) viruses implicated in the causation of benign papillomata, also known as warts. There are approximately 40 types of warts that specifically infect the anogenital region. Based on their oncogenic potential, they are divided into low- and high-risk groups [1–3]. High-risk HPV can lead to dysplasia and carcinogenesis, which accounts for 99.7% of cases of cervical squamous cell cancer, with HPV genotypes 16 and 18 being responsible for 70% of cases [4, 5]. Low-risk HPV, especially HPV genotypes 6 and 11, are the primary cause of 90% of cases of genital warts and respiratory papillomatosis [6]. Genital warts are a type of sexually transmitted disease (STD) with a high recurrence rate. Treatments for this condition include conservative and destructive regimens. Conservative therapies include agents such as imiquimod, podophyllin, podofilox, trichloroacetic acid, salicylic acid, and c. Destructive therapies, which are often the preferred treatment because they can remove lesions in only one session, include electrodessication, laser therapy, and cryotherapy [7]. While the results are usually good, these treatments are associated with pain and scarring risk; moreover, they have a significant recurrence rate [8, 9].

In recent decades, the role of zinc as an immunological modulator that activates lymphocytes and cytokines against the penetration and replication of viruses has increasingly been recognized. Patients with cutaneous warts, a benign proliferation of the skin due to low-risk HPV, were reported to have a low serum zinc level, and a high dose of oral zinc sulfate has shown outstanding therapeutic efficacy and relapse prevention [10]. To the best of our knowledge, there has been no study comparing serum zinc level in patients with genital warts and healthy people or investigating the association between serum zinc concentration and severity and recurrence of the disease. The current study was conducted to determine the zinc serum concentration in the patient population of Ho Chi Minh City Hospital of Dermatovenereology in Vietnam and identify its relationship with the clinical manifestations and presentations of genital warts. This research is expected to contribute to our understanding of the role of zinc in HPV infection.

## 2. Materials and Methods

A case-control study with convenience sampling was conducted. Patients with a clinical diagnosis of genital warts from January 2019 to July 2019 were included in this study. The habitation, profession, and educational background of the patients were analyzed. The history of the patients, including the duration (weeks) of the disease, other STDs, and previous treatments, was considered. We also clinically examined the patients, including the number and types of warts and the size and distribution of lesions. The spread of the disease, based on the total surface area of all the lesions, was defined as “mild” (<1 cm^2^), “moderate” (1–2 cm^2^), and “severe” (>2 cm^2^). The sites of the lesions including glans, shaft, anal, meatus, and perineum in male patients and vulva, vagina, meatus, cervix, and perineum in female patients were examined. A serum zinc level below 70 *μ*g/dL was considered low. Patients who had had genital warts for more than three months, despite appropriate conventional treatments, were classified as a “recurrence” group.

The exclusion criteria were as follows: patients who had taken zinc-containing products in the last three months; pregnancy; patients currently receiving treatment engage in unsafe sexual behaviors, such as having sex with more than one partner or not using condoms during intercourse; body mass index (BMI) below 18.5; human immunodeficiency virus (HIV) infection; systemic illnesses; strict diets; or any diseases involving zinc deficiency, such as atopic dermatitis, hair loss, and acne. The control group consisted of healthy individuals who were matched for age and sex. Up to 2 mL of peripheral blood was collected and then subjected to atomic absorption spectrophotometry to determine the serum zinc level. Atomic absorption spectrophotometry has been used for over 40 years to measure minerals in serum [11].

The study was approved by the Board of Ethics of the University of Medicine and Pharmacy at Ho Chi Minh City, Vietnam (447/ĐHYD–HĐĐĐ), and the authors obtained informed consent from the participants. The procedures followed were in accordance with the ethical standards of the responsible institutional committee on human experimentation as well as the Helsinki Declaration of 1975, as revised in 2013.

For statistical analysis, the data were analyzed using the STATA software (version 13). The difference of means between normally distributed groups was assessed by analysis of variance (ANOVA) and the Student's *t*-test. Furthermore, the Mann-Whitney *U* test was used to compare the differences between the two groups that were not normally distributed. The correlation between the dependent variables was assessed using Pearson's correlation. A *P* value < 0.05 was considered significant.

## 3. Results

A total of 78 patients and 78 controls (41 males and 37 females in each group) were enrolled in this study. The mean ± standard deviation (SD) value of the patients' age was 32.37 ± 9.72 years, while the mean ± SD value of the controls' age was 31.62 ± 9.29 years. Both groups showed no statistically significant difference in age or sex. The characteristics and clinical manifestations of patients are described in Table 1.

**Figure 1:**
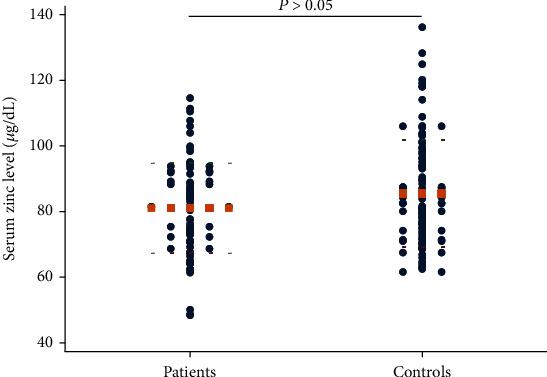
Comparison of the serum zinc levels between the patient group and the control group (Student's *t*-test). The round dots show values of serum zinc level, and the orange square dot lines show mean values.

Only 18% (14 cases) of the patients in the genital wart group and 17% (13 cases) of the healthy controls had low serum zinc levels (<70 *μ*g/dL); this difference was insignificant (*P* > 0.05). The serum zinc level in patients with genital warts had a mean ± SD value of 81.83 ± 13.99 *μ*g/dL. Meanwhile, the mean ± SD value of the serum zinc concentration in the control group was 86.66 ± 17.58 *μ*g/dL. While the patients tended to have a lower zinc concentration in the serum compared to controls, the difference between the two groups was insignificant (*P* = 0.06; Figure 1).

Following this, we analyzed the relationship between the serum zinc level and the disease duration taken from the patients' history by comparing the serum zinc levels of patients who had genital warts for less than three months with those of other patients and the control group. The same analysis was conducted with six-month and nine-month durations. However, no statistical difference was observed (*P* > 0.05; Table 2). Next, the serum zinc levels of patients with different habitations, professions, and educational backgrounds were compared; the results did not reveal significant differences (data not shown).

**Figure 2:**
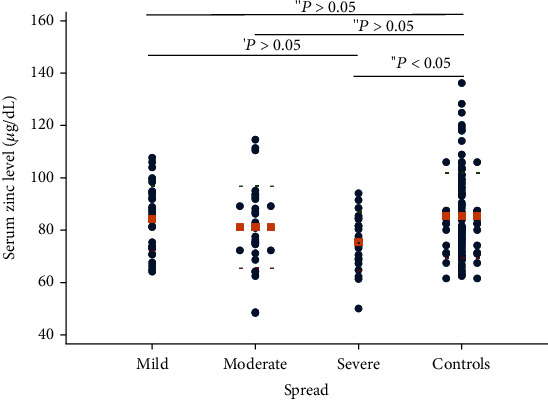
The relationship between the mean serum level of zinc and the spread of genital warts (ANOVA test; Student's *t*-test). The round dots show values of serum zinc level, and the orange square dot lines show mean values.

Regarding the spread, the mean serum zinc level of the severe subgroup was statistically different from that of the healthy controls (*P* < 0.05; Figure 2). Furthermore, there was a correlation between the serum zinc concentration and the severity of genital warts. The serum zinc levels were lower in moderate and severe patients compared to those of patients with mild lesions; however, these results were not statistically significant (*P* > 0.05; Figure 2).

**Figure 3:**
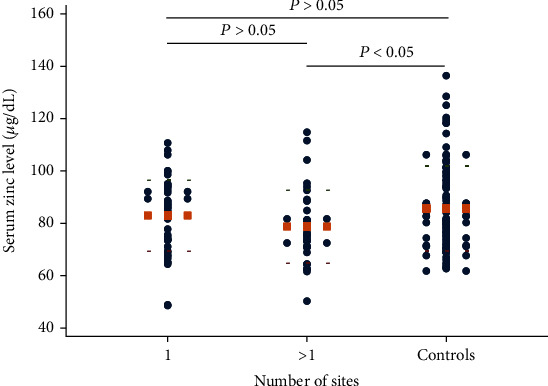
The serum level of zinc in relation to the number of sites affected (Student's *t*-test). The round dots show values of serum zinc level, and the orange square dot lines show mean values.

**Figure 4:**
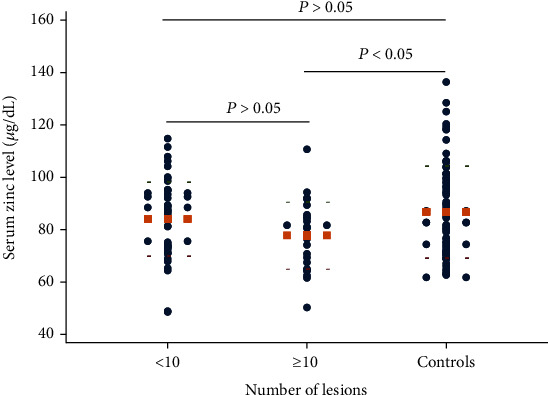
The serum level of zinc in relation to the number of lesions (Student's *t*-test). The round dots show values of serum zinc level, and the orange square dot lines show mean values.

Notably, patients with more than one affected area had significantly lower serum zinc levels in comparison to those of the controls (*P* < 0.05; Figure 3). Furthermore, the serum zinc concentration seemed to correlate with the areas affected, as patients with more affected areas had a lower serum zinc level, but no significant difference was found between the two subgroups (*P* > 0.05; Figure 3). The same outcome was observed in the variable “number of lesions” (Figure 4). Finally, in terms of recurrence, there was no correlation between serum zinc concentration and the number of relapses (data not shown).

## 4. Discussion

Genital warts, also known as condyloma acuminata, are a common dermatovenereological disease caused by HPV, a DNA virus from the *Papillomaviridae* family. The prevalence of genital warts varies between countries and geographical areas. In Canada from 1999 to 2006, the incidence ranged from 131 to 154 per 100,000 people in males and from 120 to 121 per 100,000 people in females [12]. More than 100 genomes of HPV types have been completely characterized [13]. HPV is categorized into three groups according to its potential carcinogenicity: high risk, low risk, and probably/possibly high risk [14]. HPV infection, especially types 16 and 18, is responsible for several malignant tumors, including cervical, vulvar, vaginal, penile, anal, and pharyngeal cancers [15]. The current treatment for this condition involves the physical destruction of the infected cells; however, none of these treatments are uniformly effective or directly antiviral. Several studies have concluded that systemic immunosuppression correlates with the severity of genital warts [16–18]. Moreover, Le Poole et al. found that HPV induces local immunosuppression via several mechanisms, such as inhibiting IL-10 expression, preventing the presentation of viral antigens expressed within the cell through major histocompatibility complex class I, and reducing the epithelial expression of carbonic anhydrase IX [19], which may lead to local dissemination of the lesions. According to A. Nofal et al., intramuscular or intralesional administration of a bivalent HPV vaccine resulted in complete clearance of warts [20]. The explanation could be that the HPV vaccine enhances the immune response by increasing the levels of IL-2, TNF-*α*, and proinflammatory cytokines (IL-6, IL-1*α*, and IL-1*β*), which play a crucial role in the eradication of HPV [21].

Zinc is a trace element that is essential for growth and development at all stages of life. In addition, it plays a key role in homeostasis, immune function, oxidative stress, apoptosis, and aging. Various significant disorders that are of great interest in public health are associated with zinc deficiency, including atherosclerosis, several cancers, and autoimmune diseases [22]. Regarding the immune system, zinc is essential for the normal development and function of cell-mediated innate immunity, such as neutrophils, natural killer cells, and macrophages; it impacts and collaborates specifically with all of them. Phagocytosis, intracellular killing, cytokine production, and the growth and activity of T and B cells are all affected by zinc deficiency [23].

It has been demonstrated that zinc deficiency reduces the lytic activity of natural killer cells, damages natural killer T cell cytotoxicity and immune signaling, affects the neuroendocrine-immune pathway, and adjusts cytokine production in mast cells [24, 25]. Furthermore, adaptive immune system activity depends on zinc, especially T lymphocytes. Thymulin, a hormone secreted by thymic epithelial cells, is essential for the differentiation and maturation of T cells, which require zinc as a cofactor to remain active. Without zinc, the functions of T helper type 1, the lymphocyte responsible for antivirus operation, deteriorate [22].

In our study, a low serum zinc level (<70 *μ*g/dL) was observed in 18% of patients; this was not compatible with the result of a previously published study (2010) in which 58% of patients showed a low serum zinc concentration [26]. Al-Gurairi et al. and Sadighha have reported an association between low serum zinc levels and cutaneous warts; patients with severe cutaneous warts were found to have a lower level of zinc compared to the healthy volunteers [10, 27]. In our study, we could not find any significant differences in the serum zinc level between the patients and the healthy controls. Furthermore, in terms of duration, there was an insignificant difference between the serum zinc levels of patients with disease duration longer than three months, six months, and nine months and other patients; a similar result was obtained after comparing these levels with those of the control group.

None of these findings are compatible with those of any previous studies [28, 29]. We believe this may be due to the fluctuation of the serum zinc level, different dietary customs, and our small sample size. Most of the studies we have mentioned were carried out in Iran and Iraq—Middle Eastern regions where zinc deficiency could result from the diet, hot climate, poverty, and hookworm rather than HPV infection [30]. In the Middle East, the population has diets deficient in zinc because their main food resource is cereal, which contains phytate, an inhibitor of zinc absorption; the low intake of animal protein is another contributing factor. Moreover, hot climates result in considerable loss of zinc due to excessive sweating, and the development of hookworm infection leads to blood loss; these also cause zinc deficiency. Poverty and poor healthcare facilities in these countries are other factors contributing to zinc deficiency [31]. According to Mun et al., after evaluating the serum zinc levels in 31 patients with cutaneous warts and 40 age- and sex-matched healthy volunteers, no significant difference was found between the two groups; this is compatible with our study's outcome [26]. The reason for this similarity could be that Mun et al.'s study was conducted in Japan, where the main diet is not cereal, the weather is not extremely hot, and the living standards are high, which is the case in Vietnam as well.

Another explanation for our contrary results is that all the studies cited above evaluated the serum zinc level in patients with cutaneous warts rather than genital warts [10, 27]. The difference between these two diseases makes conclusive statements regarding zinc levels difficult, and one cannot simply extrapolate findings of cutaneous warts to this group. To the best of our knowledge, only one study of Wiraguna et al. has evaluated the serum zinc level in condyloma acuminata patients, which showed that the concentration of zinc was low (<70 *μ*g/dL) regardless of HIV infection [32]. While we could not find statistically significant associations, in our cohort of patients, the serum zinc levels tended to be lower than those of the control group, and interestingly, patients with severe genital warts had lower serum zinc levels than those in control group, which is in line with the study we mentioned above. In that study, HIV-negative patients with severe genital warts had low serum zinc level (<70 *μ*g/dL). More research must be conducted to gain more clarity regarding the relationship between zinc levels and genital warts.

In terms of severity, in our study, patients with more than one involved site or large affected size (>2 cm^2^), as well as those with ten or more lesions, had a significantly lower value of serum zinc level than the controls, indicating a possible link between the importance of zinc as an immunomodulator involved in the extensiveness and severity of genital warts. This observation is in accordance with the previous studies, which indicated that a decreased serum zinc level is associated with multiple cutaneous warts [10, 27]. This could be explained by the function of zinc in the immune system. Thus, a low serum zinc level may be a good marker of dysregulated immune status. Consequently, clinical physicians should be aware of the importance of a low serum zinc level in patients. Furthermore, the data of some clinical trials support the benefit of zinc in the treatment of recalcitrant warts. In a double-blind case-control trial, 71.8% of patients treated with oral zinc sulfate recovered completely after two months of treatment, and after six months of follow-up, no instance of recurrence was observed in those who had received oral zinc therapy and responded to the treatment. Meanwhile, improvement was observed in only 13% of the control patients [33]. Akhavan et al. showed that the relapse rate in patients with vulvar warts who had been treated with oral zinc sulfate was significantly lower than that in the control patients after six months [34]. In our study, the serum zinc levels of patients with and without a history of relapse showed no statistical difference. A longitudinal study is needed to access the relationship between serum zinc concentration and recurrence.

Although we excluded patients with HIV infection, systemic illnesses, strict diets, or any diseases involving zinc deficiency, such as atopic dermatitis, hair loss, and acne, we could not evaluate a number of other underlying conditions which may relate to zinc insufficiency. In addition, some studies showed that the prevalence of zinc deficiency is aggravated by low economic status [35, 36]. However, in our study, we could not find significant differences in serum zinc levels between patients with different habitations, professions, and educational backgrounds. This could be because our study was conducted in patients in a hospital but not in community.

## 5. Conclusion

To the best of our knowledge, this is the first study regarding the relationship between the serum zinc level and the severity and extent of genital warts in a matched case-control study. The results showed that a lower serum zinc level is associated with the severity and extent of genital warts and that treatment with zinc supplements for patients with genital warts should be further investigated.

## Figures and Tables

**Table 1:** The clinical characteristics of patients with genital warts.

Clinical characteristics	Male (*n* = 41)	Female (*n* = 37)	Total (*n* = 78)	*P*
Duration (q1, q3, wk)	16 (4–25)	7.5 (4–16)	8 (4–22)	0.13^∗^
Recurrence, *n* (%)	27 (66%)	14 (38%)	41 (53%)	0.01^†^
Number of lesions (q1, q3)	6 (3 – 9)	8 (4–11)	7 (4–10)	0.07^∗^
Sites of lesions (%)	Glans, 61%	Vulva, 89%		
	Shaft, 24%	Vagina, 24%		
	Anal, 24%	Cervix, 35%		
	Meatus, 20%	Meatus, 14%		
	Perineum, 10%	Perineum, 5%		
Number of sites, *n* (%)				0.19^†^
1	27 (66%)	19 (51%)	46 (59%)	
>1	14 (34%)	18 (49%)	32 (41%)	
Spread, *n* (%)				0.41^†^
Mild	18 (44%)	13 (35%)	31 (40%)	
Moderate	16 (39%)	13 (35%)	29 (37%)	
Severe	7 (17%)	11 (30%)	18 (23%)	
Number of lesions, *n* (%)				0.08^†^
<10	31 (76%)	21 (57%)	52 (67%)	
≥10	10 (24%)	16 (43%)	26 (33%)	

q1: 1st quartile; q3: 3rd quartile; wk: weeks; ∗: Mann-Whitney *U* test; †: chi-squared test. There was a significant difference in recurrence between male and female patients (*P* = 0.01, chi-squared test), but not in the number of sites affected, number of lesions, or the spread.

**Table 2:** The association between the serum zinc level and the duration of the disease (ANOVA test).

Disease duration (*n*)	Serum zinc level (*μ*g/dL) (mean ± SD)		*P*
	Patients (*n* = 78)	Controls (*n* = 78)	
<3 months (*n* = 38)	81.01 ± 15.97	86.66 ± 17.58	>0.05
≥3 months (*n* = 40)	82.60 ± 11.96		
<6 months (*n* = 58)	81.48 ± 14.71	86.66 ± 17.58	>0.05
≥6 months (*n* = 20)	82.84 ± 11.93		
<9 months (*n* = 68)	81.71 ± 14.53	86.66 ± 17.58	>0.05
≥9 months (*n* = 10)	82.63 ± 10.03		

There was no significant difference between serum zinc levels of patients with different disease durations and healthy people (*P* > 0.05, ANOVA test).

## Data Availability

All data relating to the findings of this study are available from the corresponding author on request.
